# Experimental Study of Multispectral Characteristics of an Unmanned Aerial Vehicle at Different Observation Angles

**DOI:** 10.3390/s18020428

**Published:** 2018-02-01

**Authors:** Haijing Zheng, Tingzhu Bai, Quanxi Wang, Fengmei Cao, Long Shao, Zhaotian Sun

**Affiliations:** 1 School of Optics and Photonics, Beijing Institute of Technology, Beijing 100081, China; zhjsea08@163.com (H.Z.); liuba@bit.edu.cn (F.C.); shaolong@bit.edu.cn (L.S.); 2120150546@bit.edu.cn (Z.S.); 2 System Division, Navy Equipment Research Institute, Beijing 100161, China; ccwangok@sina.cn

**Keywords:** UAV, infrared radiation, exhaust plume, spectra

## Abstract

This study investigates multispectral characteristics of an unmanned aerial vehicle (UAV) at different observation angles by experiment. The UAV and its engine are tested on the ground in the cruise state. Spectral radiation intensities at different observation angles are obtained in the infrared band of 0.9–15 μm by a spectral radiometer. Meanwhile, infrared images are captured separately by long-wavelength infrared (LWIR), mid-wavelength infrared (MWIR), and short-wavelength infrared (SWIR) cameras. Additionally, orientation maps of the radiation area and radiance are obtained. The results suggest that the spectral radiation intensity of the UAV is determined by its exhaust plume and that the main infrared emission bands occur at 2.7 μm and 4.3 μm. At observation angles in the range of 0°–90°, the radiation area of the UAV in MWIR band is greatest; however, at angles greater than 90°, the radiation area in the SWIR band is greatest. In addition, the radiance of the UAV at an angle of 0° is strongest. These conclusions can guide IR stealth technique development for UAVs.

## 1. Introduction

With their advantages of high flexibility, low cost, high survival rate, and low operational requirements, unmanned aerial vehicles (UAVs) are widely used in civil fields such as urban management, agricultural monitoring, environmental protection, disaster relief, and film and television photography [1,2,3,4]. In the military fields of surveillance, monitoring, electronic confrontation, and damage assessment, UAVs play an important role as well [5,6]. The target drone is a kind of UAVs that has important applications in the military field [7,8]. As the imaginary target of various artillery or missile systems, the target drone should have some a certain stealth performance. Furthermore, infrared (IR) radiation features is the major factor for stealth performance. For this reason, studies on the infrared (IR) radiation characteristics of the target drone are of great significance to IR stealth techniques regarding target drone’s body, engine, and fuel formulation design and to IR warning system development. Generally, the power plant of a UAV with long endurance is commonly a turbo fan engine. For the target drone and other small high-speed aircraft, such as unmanned reconnaissance aircraft, a gas turbine engine is used as its propeller.

A number of IR radiative sources impinge on UAV elements such as its surface, engine, and exhaust plume. The IR radiation emitted from the exhaust plume is one of the main analysis targets in IR steath technology. Many studies have investigated the IR radiation of both an aircraft and its exhaust plume by numerical simulations and/or experimental methods. The IR characteristics of a typical commercial quad-rotor UAV were investigated through thermal imaging with an IR camera by Gong et al. [9]. Blunck et al. measured the narrowband radiation intensity of exhaust plumes exiting from a converging nozzle with varying Reynolds numbers, Mach numbers, temperatures, and species compositions corresponding to low fuel-to-air equivalence ratios [10]. For the test conditions studied, the intensity emanating from diametric paths first decayed linearly with increasing distance from the nozzle exit and then decayed exponentially with an inflexion point near the end of the plume core. In 2014, a concise band selection method employing the multispectral signatures of stealth aircraft was proposed by Liu et al. [11]. The stability of the selected bands was tested under varying environments. The key step in the model was to select two or more optimal bands that could clearly signify the radiation difference between the target and its background. Two narrow bands of 2.86–3.3 μm and 4.17–4.55 μm were ultimately selected after detailed analyses on contrast characteristics between the target and background. In 2016, Huang studied the plume IR radiation of a long-endurance UAV [12] and found that the IR radiation energy of the plume was distributed mainly at the IR wavelengths of 2.7 μm and 4.5 μm. In 2017, a multi-scale narrowband correlated-k distribution (MSNBCK) model has been developed by Zhou et al. [13] to simulate IR radiation from the exhaust system of a typical aircraft engine. In the model, an approximate approach significantly reduces the required computing power by converting the exponential increase of computations required with the increase of participating gas species to a linear increase. The results indicate that a wall’s IR emission should be considered in both 3–5 μm and 8–14 μm ranges while the IR emission of a gas plays an important role only in the 3–5 μm band. They also found that carbon dioxide’s IR emission is much more significant than that of water vapor in both the 3–5 μm and 8–14 μm bands. In particular, in the 3–5 μm band, water vapor’s IR signal can even be neglected compared with that of carbon dioxide. In 2017, Retief et al. measured a plume in short-wavelength infrared (SWIR) (1.1–2.5 μm), mid-wavelength infrared (MWIR) (2.5–7 μm), and long-wavelength infrared (LWIR) (7–15 μm) cameras and a spectral radiometer covering the entire mid, long, and upper part of the short-wavelength infrared bands [14]. The experimental results showed that the MWIR band was the most versatile regarding plume observations. The fuel consumption of an aircraft and the observation configuration were found to be the main influences on the detected emission of an aircraft plume.

Few studies have concentrated on the IR features of a target drone and the IR difference between a UAV fuselage and its engine. The research presented here will be helpful for the IR stealth performance of the target drone. Nevertheless, the methodologies used in previous studies are the references to this research. In this paper, the measurements of both a target drone and its gas turbine engine in different IR bands within the 0.9–15 μm band are shown. SWIR, MWIR, and LWIR cameras are used to obtain IR images of the targets. The IR spectrum is obtained by a spectral radiometer cooled by liquid nitrogen. By rotating the UAV or its engine, the observation angle between the target and the observer changes from 0° to 180°. Subsequently, IR characteristics of the complete UAV and its engine at different observation angles are recorded by the instruments mentioned above. The complete UAV and its engine operating in cruise on the ground are tested separately. The results show that the difference in IR characteristics between the complete UAV and its engine are very important for detecting a UAV when other IR sources are present, such as IR jamming. The results are also significant for the IR stealth design of UAVs.

## 2. Materials and Methods

### 2.1. Measuremental Layout

**Figure 1:**
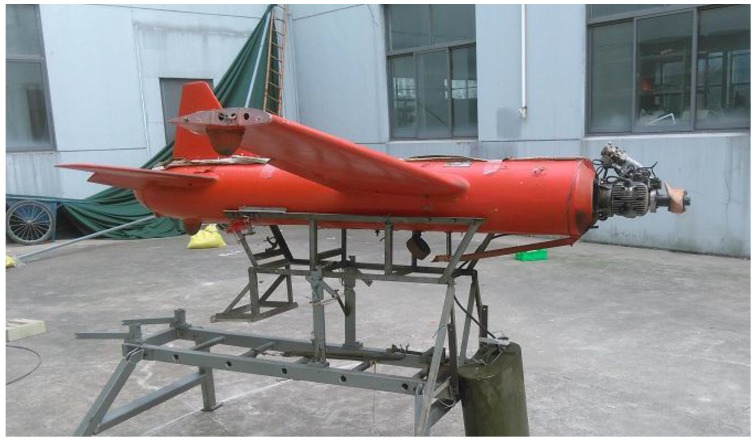
The unmanned aerial vehicle (UAV).

**Figure 2:**
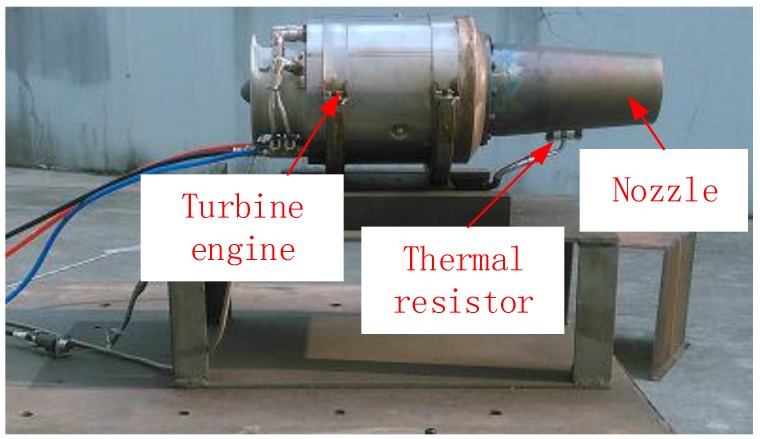
The turbine engine.

The tested UAV was a target drone in the research and development phase. Its power plant was a turbine engine with a shrinkable nozzle with inlet and outlet diameters of 96.5 and 72.1 mm, respectively, as shown in Figure 1 and Figure 2. Beneath the nozzle on the wall was a thermal resistor that was used to measure temperature. The engine burned aviation kerosene as its fuel.

**Figure 3:**
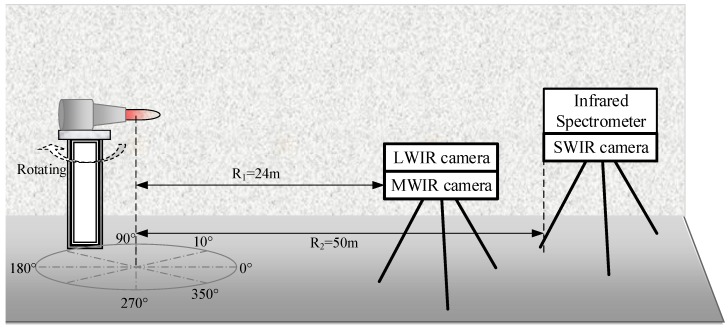
Experiment layout.

The experimental layout is shown in Figure 3. In the test, the instruments and the target (plume or complete UAV) were mounted orthogonal to the plume centerline. The target could be rotated from 0° to 180°. An observation angle of 90° meant that the viewing direction was perpendicular to the direction of the plume flow. An observation angle of 0° meant that the exhaust plume flowed directly toward the observer.

### 2.2. Radiation Measurements

A liquid-nitrogen-cooled MR170 portable IR spectral radiometer with a spectral range of 740–5000 cm^−1^ (2–13.5 μm), and a spectral resolution of 1 cm^−1^ was used in the test to obtain the spectrum. This instrument’s field of view (FoV) covered the whole area of the plume and also the complete UAV.

Integrated radiation intensity was calculated by

(1) $$I_{\Delta \lambda }=\int_{\lambda_{1}}^{\lambda_{2}}I_{\lambda }d\lambda$$

where the subscript Δ*λ* denotes the wave band, *λ*_1_ and *λ*_2_ are the band boundary respectively, and *I* is the radiation intensity.

Different bands IR images were recorded by three different IR cameras. The wavelength ranges of the LWIR, MWIR, and SWIR cameras were 7.7–9.3 μm, 3.0–4.8 μm, and 0.9–1.7 μm, respectively. The instrument specifications used in this experiment are summarized in Table 1 below.

**Figure 4:**
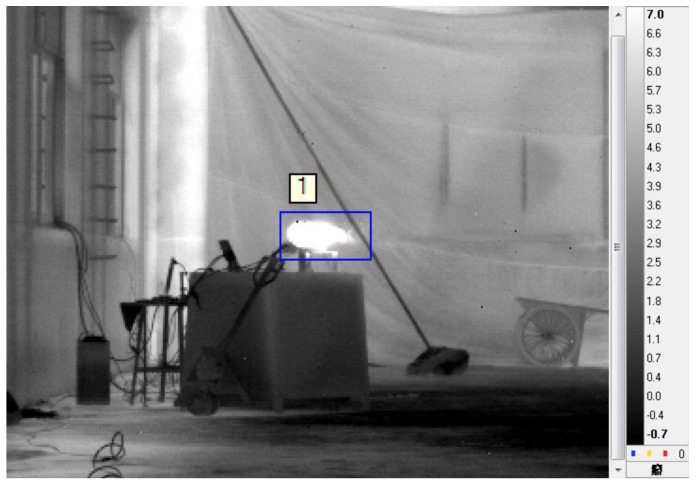
Blue frame illustration.

The radiation area of the target was obtained through processing the data. For calculating the radiation area of the plume or the complete UAV, a blue frame was marked manually in the IR images, illustrated in Figure 4.

The height and width of the blue frame were calculated by

(2) $$h=eLN_{h}/f^{\prime },w=eLN_{w}/f^{\prime },$$

where *h* is the object height, *w* is the object width, *e* is the pixel size, *L* is the operating distance, *f’* is the focal length of the camera, *N_h_* is the pixel number of the blue frame’s height, and *N_w_* is the pixel number of the blue frame’s width. The radiation area was then calculated as the product *h* × *w*.

### 2.3. Surrounding Radiation and Calibration

Since the atmosphere surrounds the UAV and IR detectors, the IR characteristics of the atmosphere play an important role in dictating the IR signatures as perceived by the detectors. The atmosphere is a good absorber of IR radiation. Radiant flux from the target aircraft is selectively absorbed by several atmosphere gases and scattered away by small particles suspended in atmosphere. Generally, sky radiance, cloud reflecting radiance, and other background radiance are also should be taken into account. In this work, the test was implemented indoors. Hence, the sky radiance and cloud radiance could be neglected. To further eliminate the effect of background radiation, a black curtain was arranged at the end of the test site.

These cameras were calibrated using an extended area blackbody. The blackbody was placed at the same distance from the camera as the plume centerline and was surrounded by the same atmosphere with the UAV. This approach was used to account for atmosphere attenuation of the IR radiation.

### 2.4. Operating Conditions

**Figure 5:**
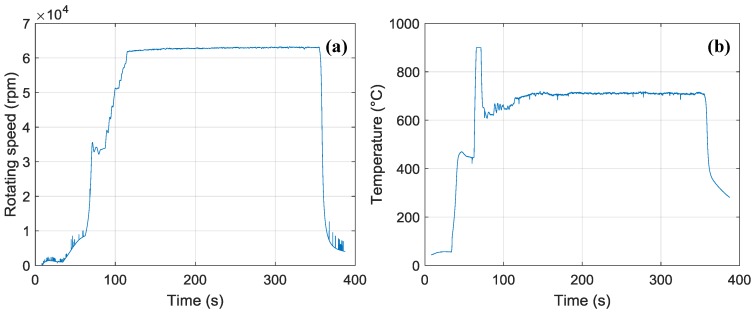
Engine rotation speed and temperature. (**a**) Time-dependent rotating speed of the engine; (**b**) Time-dependent temperature of the outlet wall.

The rotation speed and temperature of the engine were measured during the experiment as shown in Figure 5. As the turbine engine began to operate, the rotation speed and temperature increased rapidly. At the cruise stage, the rotation speed and temperature stabilized. Once operating stably, the IR spectral radiometer and IR cameras were used to obtain information from the targets at a time of about 400 s. Afterward, the engine’s rotation speed was reduced in consideration of safety, and the whole target was rotated by some angle to achieve the purpose of changing the observation angle. Subsequently, the engine was returned to the cruise stage, and instruments obtained information at the new observation angle.

## 3. Results

### 3.1. Turbine Engine

#### 3.1.1. Spectral Radiometer Recording

**Figure 6:**
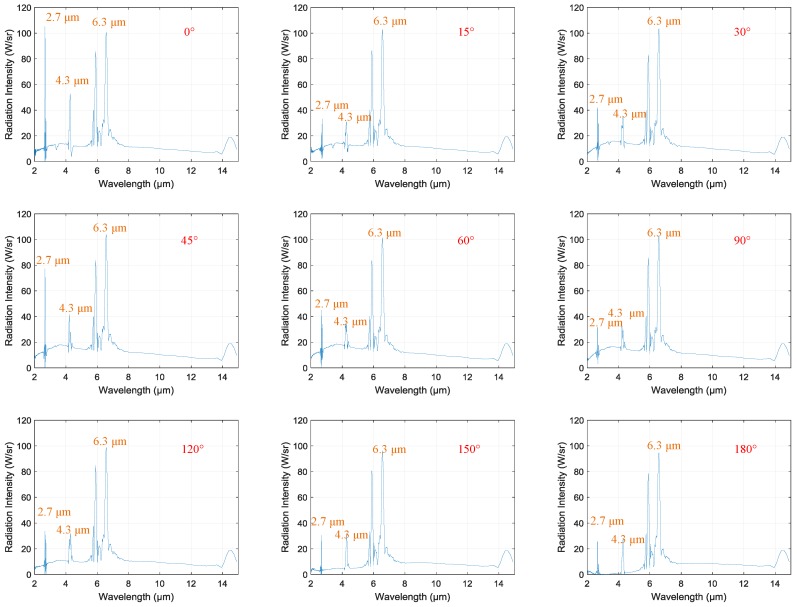
Spectral radiation intensity of the engine at different observation angles.

Figure 6 shows the spectral radiation intensity of the engine in its cruise state at different observation angles: 0°, 15°, 30°, 45°, 60°, 90°, 120°, 150°, and 180°. Several points should be mentioned in regard to this figure.

1. All spectra were continuous, with spectral bands and lines.
2. All spectra had three highlighted bands: 2.7 μm, 4.3 μm, and 6.3 μm.
3. The values of radiation intensity at 2.7 μm and 4.3 μm changed with the observation angle, but that at 6.3 μm remained unchanged.

Regarding gas molecules, each spectral band involved a change in the vibrational energy of the molecules. Lines in the spectral bands resulted from simultaneous changes in rotational energy [15]. Hence, the spectral bands and lines of spectra in Figure 6 were produced by hot gas emissions in the exhaust plume. The continuous spectra were caused by solid matter, most probably carbon soot [14]. We know that water vapor’s main emission bands occur at 2.7 μm and 6.3 μm [16], while those of carbon dioxide occur at 2.7 μm and 4.3 μm [17] within the range of 2–15 μm. Therefore, the emission bands of 2.7 μm, 4.3 μm, and 6.3 μm result from hot water vapor and carbon dioxide emissions. However, the values of radiation intensity at 2.7 μm and 4.3 μm changed with the observation angle and that at 6.3 μm remained unchanged. Therefore, emitters producing 2.7 μm and 4.3 μm bands were in the exhaust plume and emitters producing the 6.3 μm band were in the background. Considering the substantial combustion product already expelled into the air, the 6.3 μm band may have been caused by water vapor in the background.

The main bands of 2.7 μm and 4.3 μm are resulted from exhaust plume emissions and the 6.3 μm band is resulted from background emissions. According to Rao [18], IR radiation emitted from aircraft exhaust plumes arises primarily from carbon dioxide and carbon monoxide in the exhaust. The experimental results for spectral radiation intensity agree with Rao’s conclusion.

#### 3.1.2. IR Camera Recording

**Figure 7:**
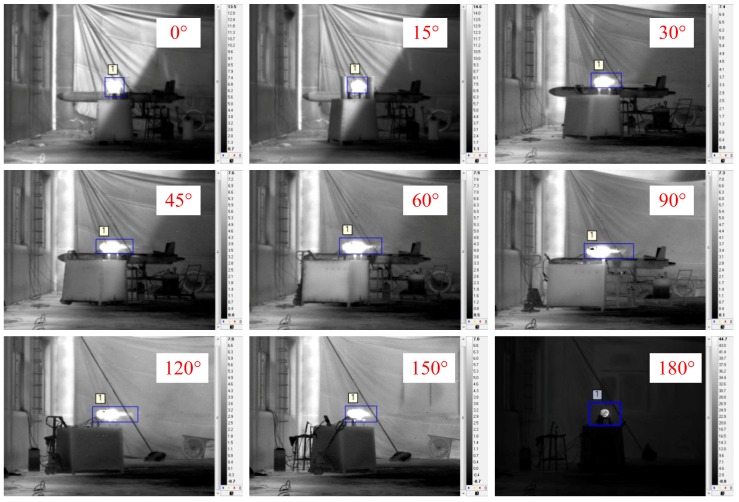
Engine images captured by the long-wavelength infrared (LWIR) camera at different observation angles.

Figure 7 shows images of the engine operating in the cruise stage captured by the LWIR camera at different observation angles. The target was relatively easy to discriminate from the background. In Figure 7, the bright area can be identified as the engine, but exhaust plume structures are difficult to distinguish.

Although the target was only the engine, the body of the drone may be recognized at some observation angles. The cause of this problem was that the turbine engine was disassembled but must remain connected to the UAV. During the test, this problem was not noticed at first. Afterward, from the observation angle of 120°, the body of the drone was removed. Nevertheless, there was a minimal effect on the radiance because of the blue frame.

**Figure 8:**
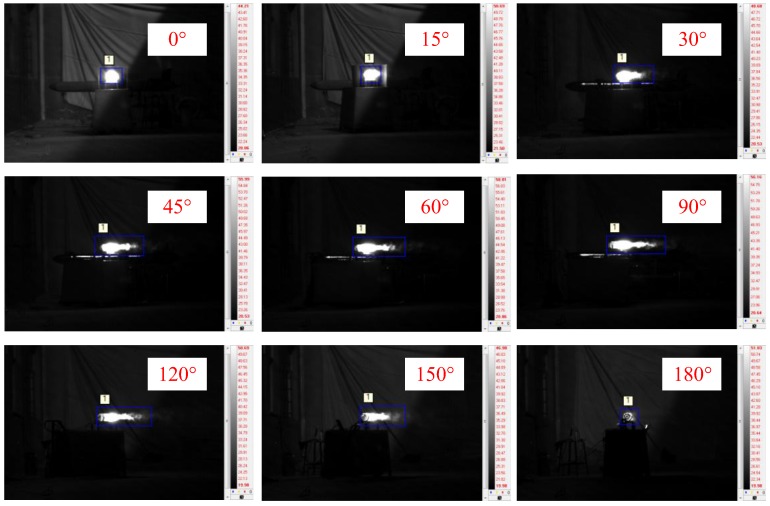
Engine images captured by the mid-wavelength infrared (MWIR) camera at different observation angles.

Figure 8 shows images of the engine operating in the cruise stage captured by the MWIR camera at different observation angles. The target was easy to discern relative to the background, and the exhaust plume structures can be distinguished clearly in the images at observation angles of 45°, 60°, 90°, 120°, and 150°. At an observation angle of 0°, the exhaust plume presented as a disc. In contrast, at an observation angle of 180°, the exhaust plume could scarcely be seen because it was blocked by the cold parts of the engine.

**Figure 9:**
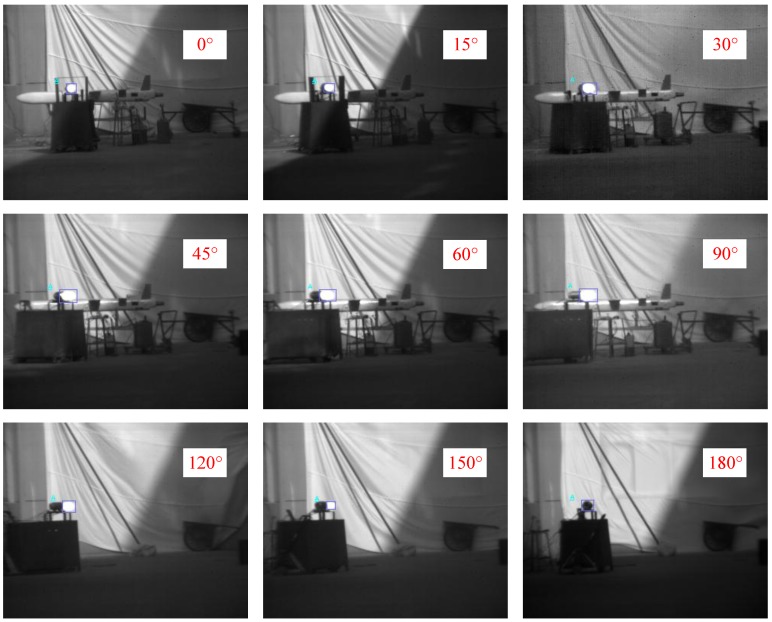
Engine images captured by the short-wavelength infrared (SWIR) camera at different observation angles.

Figure 9 shows images of the engine operating in the cruise stage captured by the SWIR camera at different observation angles. The target was difficult to discern from its background. At an observation angle of 180°, the exhaust plume could not be seen.

**Figure 10:**
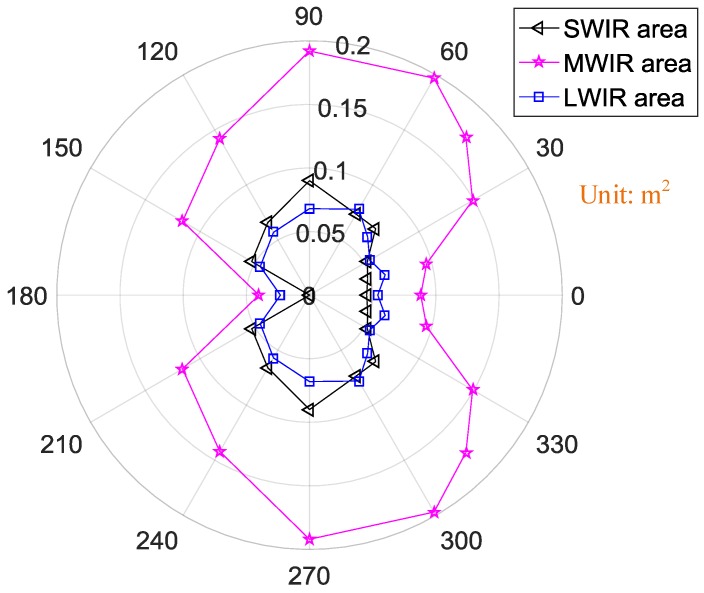
Orientation map of the engine’s radiation areas in different infrared (IR) bands.

From Figure 10, the exhaust plume along with the hot engine is relatively obvious in the MWIR band because it has the largest radiation area. The tendencies of the radiation area similarly change with the observation angles in the three IR bands. The radiation area increased with the observation angle from 0° to 90°. At an observation angle of 90°, the radiation area was largest. However, the further increase of the observation angle resulted in a rapid decrease in radiation area. The least radiation area occurred at observation angle of 180°, mostly because the hot engine and exhaust plume were blocked in that configuration by cold parts of the engine.

**Figure 11:**
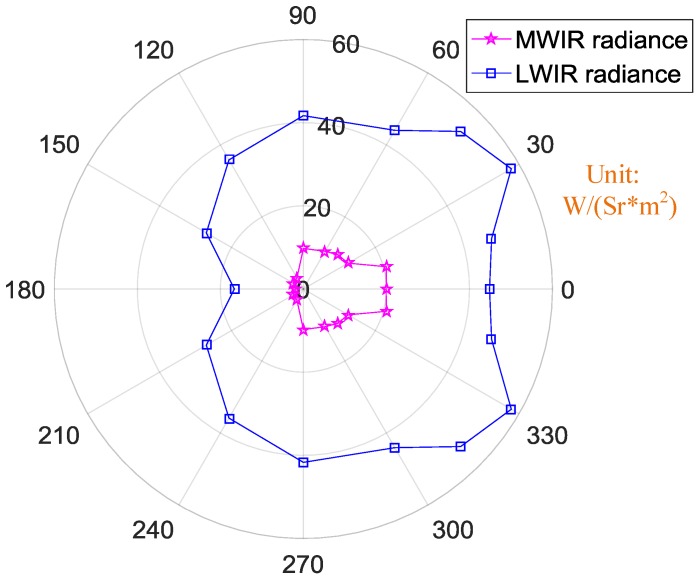
Orientation map of the engine’s radiance in different IR bands.

The radiance in different IR bands at different observation angles were also calculated, as shown in Figure 11. The exhaust plume and hot engine emitted the largest amount of radiance in the LWIR band though the smallest radiation area. Nevertheless, in LWIR band, radiance at an observation angle of 30° was the largest, almost 60 W/(Sr*m^2^). With an increasing observation angle, the radiance decreased. The least radiance in both the LWIR band and the MWIR band occurred at an observation angle of 180°. In LWIR band, the least radiance was 18 W/(Sr*m^2^), while in the MWIR band, it was almost zero.

### 3.2. Complete UAV

#### 3.2.1. Spectral Radiometer Recording

**Figure 12:**
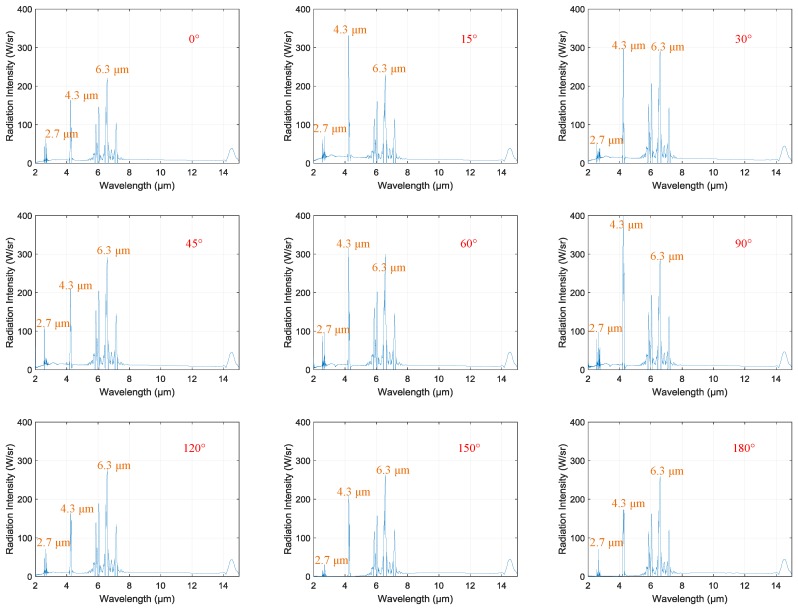
Spectral radiation intensity of the UAV at different observation angles.

The behavior of spectral radiation intensity for the complete UAV was the same as that for the engine which is shown in Figure 12. The values of radiation intensity in the 2.7 and 4.3 μm bands changed with the observation angle, but that in the 6.3 μm band remained unchanged. The only difference was in the absolute value of radiation intensity. Overall, the complete UAV’s radiation intensity value was about three times that of the engine’s. Take the observation angle of 90° for example: at an observation angle of 90° with a wavelength of 6.3 μm, the radiation intensity of the complete UAV was 298 W/sr, while the radiation intensity of the engine was 101 W/sr.

#### 3.2.2. IR Camera Recordings

**Figure 13:**
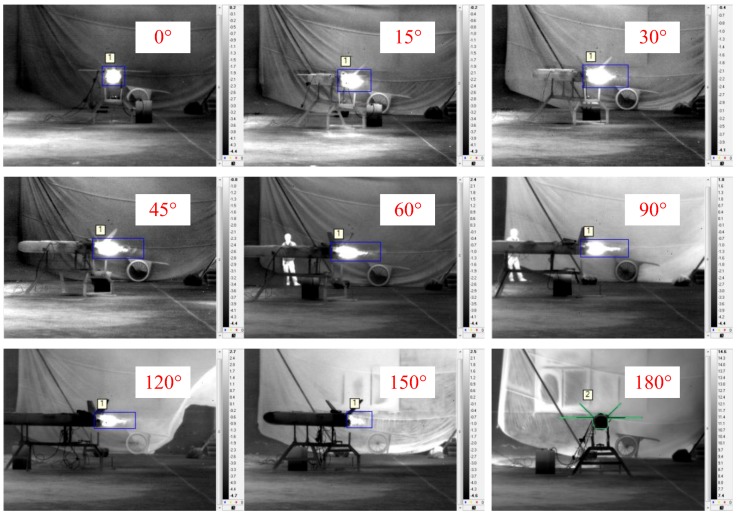
UAV images captured by the LWIR camera at different observation angles.

Figure 13 shows images of the whole UAV operating in the cruise stage captured by the LWIR camera at different observation angles. In the LWIR band, the body of the UAV emitted some radiation; however, compared to the engine and the exhaust plume, the body’s radiation level was too low.

**Figure 14:**
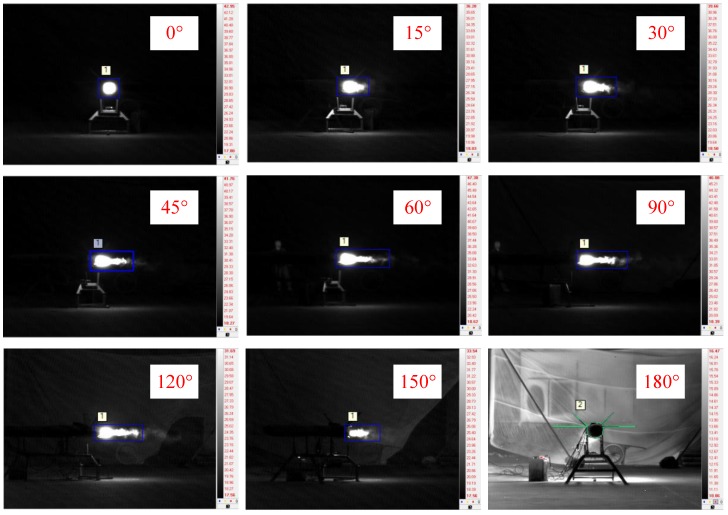
UAV images captured by the MWIR camera at different observation angles.

Figure 14 shows images of the whole UAV operating in the cruise stage captured by the MWIR camera at different observation angles. Only the hot engine and exhaust plume could be seen at observation angles from 0° to 150 °. At 180°, owing to the large body size of the UAV, the hot engine and the exhaust plume did not appear.

**Figure 15:**
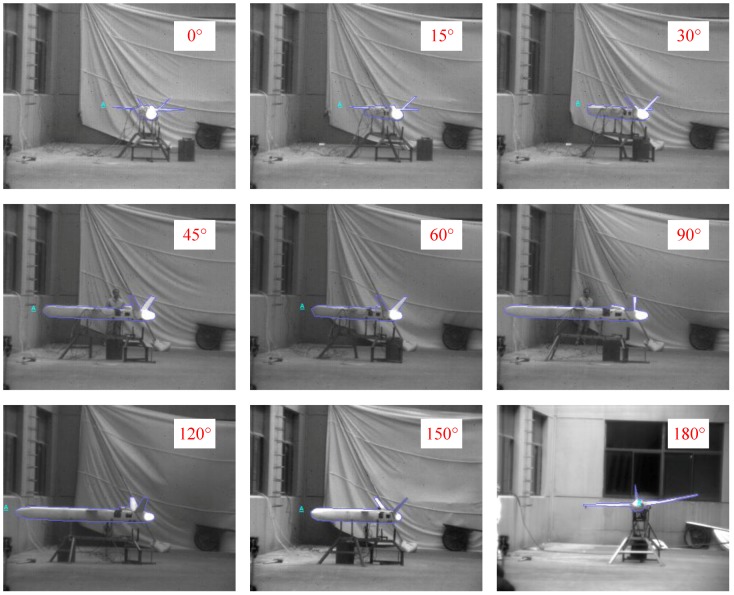
UAV images captured by the SWIR camera at different observation angles.

Figure 15 shows images of the complete UAV operating in the cruise stage captured by the SWIR camera at different observation angles. In the SWIR band, the exhaust plume was not obvious, but the body’s appearance was still relatively distinct.

**Figure 16:**
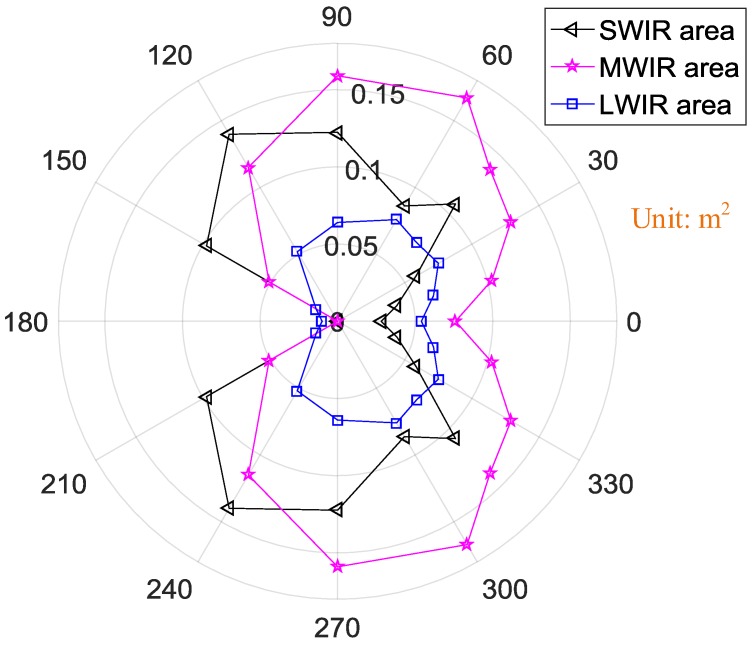
Orientation map of the UAV’s radiation areas in different IR bands.

The orientation map of the UAV’s radiation area in different IR bands is shown in Figure 16. Because of the obvious body in the SWIR band, the radiation area in SWIR band was larger than that in the MWIR band at observation angles from 120° to 150°. But the largest radiation area in SWIR band was 0.14 m^2^ at observation angle of 120° because of the very large body of the UAV, while that in MWIR band was 0.08 m^2^ at 60°. There is the least radiation area in the LWIR band.

**Figure 17:**
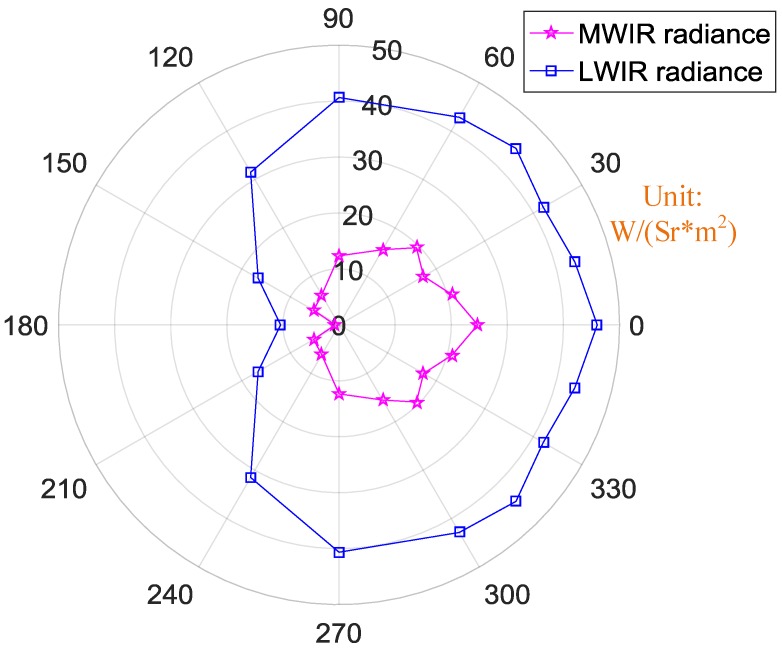
Orientation map of the UAV’s radiance in different IR bands.

The radiance of the complete UAV in different IR bands at different observation angles was calculated; the results are shown in Figure 17. The radiance values at observation angles from 0° to 90° were relatively close, about 42 W/(Sr*m^2^). The maximum radiance occurred at an observation angle of 0°, 45 W/(Sr*m^2^). When the observation angle was greater than 90°, the radiance value diminished rapidly from 40 W/(Sr*m^2^).

### 3.3. Comparison

**Figure 18:**
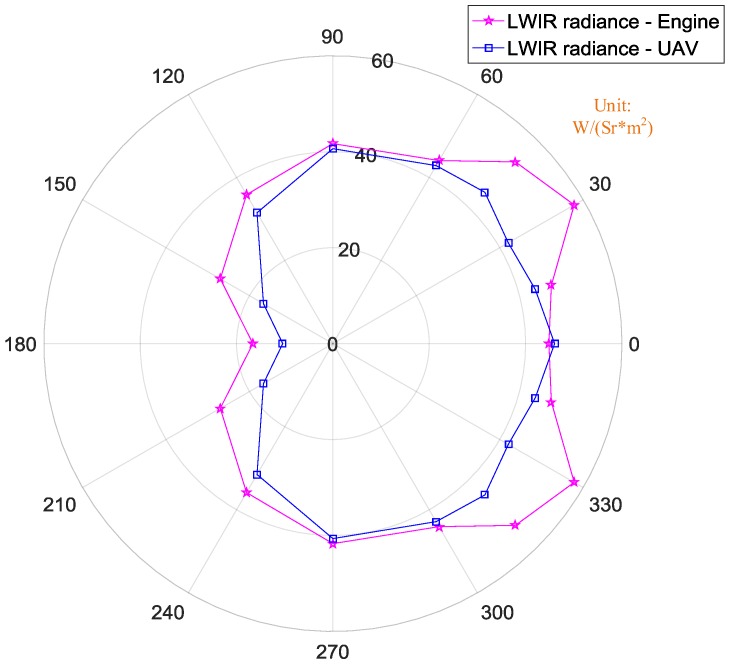
Comparison of LWIR radiance between engine and UAV.

Figure 18 shows the difference of the levels measured in terms of LWIR radiance orientation maps between the turbine engine and the complete UAV. The LWIR radiances of the turbine engine and UAV were roughly the same at different observation angles, except 30°–45°. At an observation angle of 30°, the engine’s radiance in LWIR band was 58 W/(Sr*m^2^), while the complete UAV’s radiance in LWIR band was only 41 W/(Sr*m^2^). It is worthwhile to note that the largest radiance of the engine in LWIR band is at an observation angle of 30°, that is 58 W/(Sr*m^2^). In other words, under the current structure design of the UAV, the LWIR characteristics of the engine and its exhaust plume were mainly eliminated.

**Figure 19:**
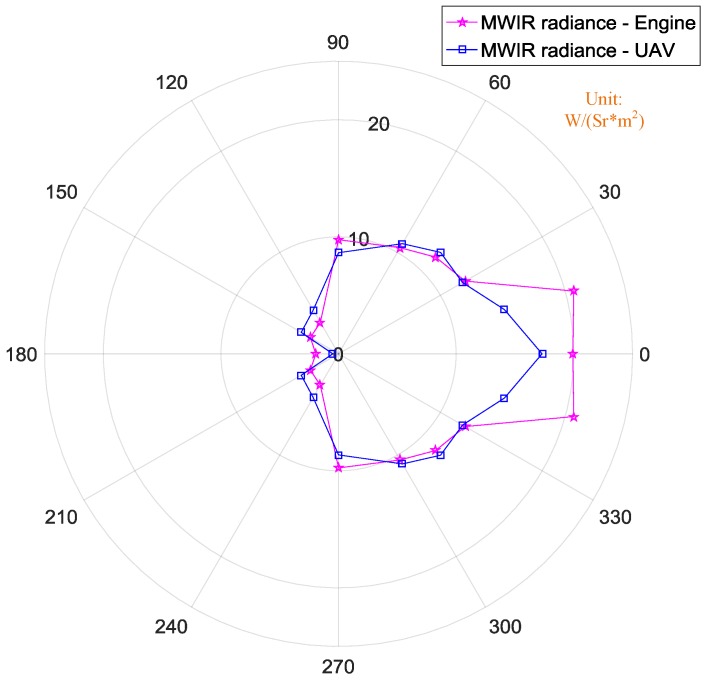
Comparison of MWIR radiance between engine and UAV.

Figure 19 shows the difference of the levels measured in terms of MWIR radiance orientation maps between the turbine engine and the complete UAV. For both the engine and the complete UAV, the radiance values in MWIR band were half that in LWIR band. In MWIR band, the largest radiance of the UAV was at 0°, which was 18 W/(Sr*m^2^). As the observation angle increasing, radiances of the engine and the UAV continuously decreased. The main difference of the MWIR radiance values between the engine and the UAV was from 0° to 30°. At other angle of 30°–180°, the radiance values were almost the same. This difference suggests that the fuselage only affects the MWIR radiance of engine and its exhaust plume from observation angles of 0–30°.

## 4. Discussion and Conclusions

In this work, the IR radiation characteristics of a complete target drone and its engine were studied experimentally. The UAV and its engine were tested in the cruise state on the ground. The spectral radiation intensity for the UAV and its turbine engine at different observation angles were obtained by a spectral radiometer, and IR images were captured by LWIR, MWIR, and SWIR band cameras separately. The orientation maps of the radiation area in different IR bands were calculated, and the orientation maps of radiance in different IR bands were obtained. From the results, the following conclusions can be drawn.

- The spectral radiation features of the UAV in IR bands of 2–15 μm is determined by its exhaust plume; specifically, by the combustion products. According to Cain, fuel chemistry affects selectivity for specific decomposition pathways, and unburned fuel components are observed in the engine exhaust plume during operation with all fuels [19]. Except for the 2.7, 4.3, and 6.3 μm bands, other lines in Figure 5 and Figure 11 may result from unburned fuel. Further research must be conducted to study the influence of other material in the exhaust plume on spectral radiation characteristics.
- IR radiation emitted from the UAV is primarily from carbon dioxide and water vapor. The main IR emission bands are at 2.7 and 4.3 μm. According to Rao [18], IR radiation emitted from a gas turbine engine exhaust plumes arises primarily from carbon dioxide and carbon monoxide. And Liu [11] found that 2.86–3.3 μm and 4.17–4.55 μm were the main emission bands determined by the two main constituents of the plume gas, namely CO_2_ and H_2_O. These conclusions are in good agreement with the current experimental results.
- At observation angles from 0–90°, the radiation area of the target drone in the MWIR band was the largest. However, at observation angles greater than 90°, the radiation area of the UAV in the SWIR band was the largest. This is important for the IR detection of target drones. When aiming at a target drone in transit, the observation angle is less than 90°. In this case, an MWIR detector may be the best choice. However, in the case of aiming at a target drone flying toward the observer, the observation angle is greater than 90°. In this case, an SWIR detector may be the best choice. But it should be noted that this conclusion has a precondition that the test is implemented on the ground indoors. During flight conditions, the drone interacts with its environment and undergoes solar illumination or background radiation from the sky and the ground. Further research under these circumstances is required.
- In the IR band, the radiance of the target drone at an observation angle of 0° is the strongest; however, regarding the engine, the radiance at an observation angle of 45° was strongest in the LWIR and MWIR bands. The radiance values of the engine at observation angles from 0° to 45° are relatively close. However, as the engine is embedded in a UAV, at an observation angle of 45°, the body of the UAV blocks most of the radiance emitted from the exhaust plume and the hot engine.

This paper provided additional and improved insight into the emission propensity in a target drone powered by a turbine engine. These conclusions are of great significance for IR stealth techniques regarding target drone’s body, engine, and fuel formulation design and for IR warning system development.

## Figures and Tables

**Table 1:** Instrument specifications.

Instrument	Model	Wavelength Range
Infrared Spectral Radiometer	AnalyzelIT MR170	2–13.5 μm
LWIR Camera	FLIR SC7300L	7.7–9.3 μm
MWIR Camera	FLIR SC7300M	3.0–4.8 μm
SWIR Camera	Xenics Bobcat-640	0.9–1.7 μm
